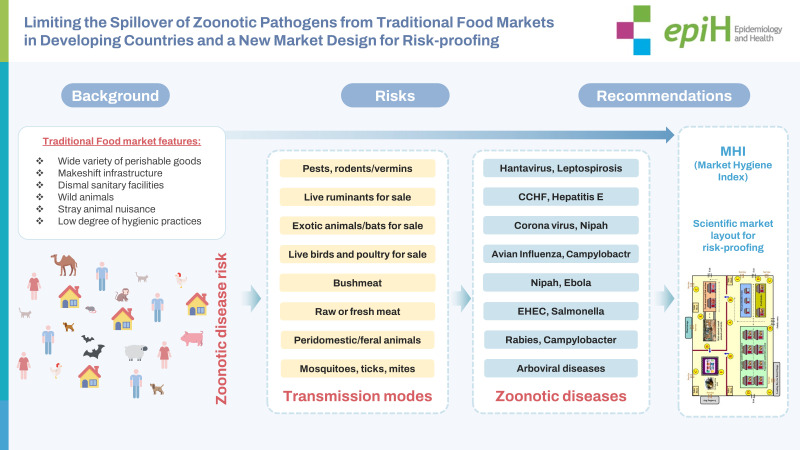# Limiting the spillover of zoonotic pathogens from traditional food markets in developing countries and a new market design for risk-proofing

**DOI:** 10.4178/epih.e2023097

**Published:** 2023-10-30

**Authors:** Sandeep Ghatak, Kandhan Srinivas, Arockiasamy Arun Prince Milton, Govindarajan Bhuvana Priya, Samir Das, Johanna F. Lindahl

**Affiliations:** 1 Division of Animal and Fisheries Sciences, ICAR Research Complex for NEH Region, Umiam, India; 2 Division of Veterinary Public Health, ICAR-Indian Veterinary Research Institute, Izatnagar, India; 3 College of Agriculture (Kyrdemkulai), Central Agricultural University, Imphal, India; 4 International Livestock Research Institute, Hanoi, Vietnam; 5 Department of Medical Biochemistry and Microbiology, Uppsala University, Uppsala, Sweden

**Keywords:** One health, Wildlife trade, Hygiene, Policy, Strategic planning, Evidence-based facility design

## Abstract

Traditional food markets are age-old systems that primarily serve the food supply needs of society’s less affluent sectors, often operating with minimal infrastructure. These markets are prevalent in low and middle-income countries. However, their hygienic conditions are frequently suboptimal, potentially fostering the emergence and spread of presumptive zoonotic diseases. The recent emergence of zoonotic or potentially zoonotic diseases and their possible links to traditional food markets underscore the need for focused attention on this overlooked issue. The socioeconomic characteristics of traditional food markets reveal that despite the risk of zoonotic pathogen spread, these markets play a crucial role for large segments of the population. These individuals rely on such markets for their livelihood, food, and nutrition. Therefore, a comprehensive set of measures addressing various aspects of traditional food markets is necessary to manage and mitigate the risks of potential zoonotic disease emergence. In this article, we explore various facets of traditional food markets, paying special attention to the risks of zoonotic diseases that urgently require stakeholder attention. We also propose a new market design to prevent the risk of zoonotic spillover and advocate for the development of a Market Hygiene Index for these markets.

## GRAPHICAL ABSTRACT

[Fig f3-epih-45-e2023097]

## INTRODUCTION

A “traditional food market” is a term broadly used to describe open-air markets that provide a variety of food items to meet people’s daily needs. These markets serve as informal retail points within the value chains of various agricultural and animal products. Traditional markets fulfill the daily requirements for fresh and affordable foods for millions, particularly in low and middle-income countries (LMICs). Furthermore, these markets are deeply integrated into the social fabric of communities, offering a space for social interaction and networking within the informal sector. They also cater to the needs of low-income groups and minority sections of society [1,2].

Globally, the largest share of agricultural produce is sold through informal and traditional markets, particularly in LMICs. These traditional informal markets play a vital role in providing food security and nutrition for large segments of the population in Asia and Africa, particularly attracting customers from marginalized societal groups [3,4]. For instance, data from Vietnam indicated that approximately 95% of Hanoi residents’ vegetable needs were met by around 400 traditional markets [5,6]. Similarly, in African countries such as Tanzania, Kenya, Uganda, Rwanda, Ethiopia, and South Sudan, between 86% and 99% of milk sales took place through informal channels [7]. Therefore, it is reasonable to expect that the majority of food and related product trading takes place in these traditional markets.

Although traditional markets are integral to the economy, particularly in LMICs, due to their inherent nature, they present significant public health risks. These risks include food safety concerns, the spread of communicable diseases, and the emergence of zoonotic diseases. The latter concern was underscored during the coronavirus disease 2019 pandemic, with studies suggesting the potential origin of the causative virus, severe acute respiratory syndrome coronavirus 2, in a Chinese market specializing in live animals. However, the risks associated with traditional markets have been highlighted in the past as well [8]. Other examples include the transmission of avian influenza to humans in a live poultry market in Hong Kong, and the potential emergence of the first severe acute respiratory syndrome coronavirus in 2003 due to the sale of civet cats in a traditional wet market [4,9]. Therefore, it is essential to discuss and develop strategies to mitigate the risks arising from traditional markets. However, the development of these risk mitigation strategies requires a comprehensive understanding of the risks associated with traditional markets, which are shaped by the unique characteristics of these markets.

## CHARACTERISTICS OF TRADITIONAL MARKETS

Traditional food markets exhibit immense diversity across different countries and cultures, reflecting the communities and societies they serve. While crafting a universal definition is challenging, certain characteristics distinguish traditional food markets from other types of markets, such as supermarkets. The term “wet market” is often used in conjunction with traditional markets, originating from the practice of using water or ice to keep fish and fresh meat cool, which often results in a soggy, and sometimes muddy, floor. This contrasts with “dry markets,” which typically trade in non-perishable products like clothing and electronics. For the purposes of this discussion, we will use the term “traditional food markets” to encompass “wet markets” as well [4,9–12].

(1) Traditional food markets typically offer a variety of perishable goods, catering to a broad cross-section of society or community. (2) These markets typically comprise numerous open-air stalls scattered across an area, featuring multiple entry and exit points for both buyers and sellers. The infrastructure is often inadequate, and it is common for sellers to erect makeshift shelters to protect themselves from the rain and sun in tropical climates. (3) Sanitary amenities such as waste disposal, insect/pest control measures, toilets, water supply, and drinking water facilities are often dismal. (4) *In situ* slaughter and carcass dressing of animals such as goats, sheep, fish, and birds are also a conspicuous feature of traditional wet markets. (5) Some of these markets also illegally sell wild or slaughtered animals, such as wildlife and protected/endangered animals, as has been reported in many African and Asian nations [9,11,13–15]. (6) Abundant stray animals and birds feeding on waste material are a common sight in traditional marketplaces. (7) Eatables/street foods are often sold in these markets under unhygienic conditions, mostly to serve the needs of the sellers, but also for buyers. (8) Traditional markets are “nutrient-dense” markets and are vital sources of affordable food and nutrition for communities. (9) A key feature of these markets is the freshness of the products compared to preserved, processed products on the supermarket shelves. (10) Traditional markets offer many products unique to the local culture and are often aligned with the culinary customs and practices of the region. (11) These markets are an important source of livelihood for people from poorer sections of society including traders and consumers, in both rural and urban settings. (12) Transactions in these markets are usually trust-based between retailers and consumers and are also influenced by product quality and cost. (13) Sellers in these markets are characterized by low awareness about product hygiene, low literacy rates, limited financial resources, and the absence of traditional safeguards such as insurance against business failure, rendering them reluctant to improve their hygienic practices. (14) Though these markets may operate under local regulatory bodies, they are often poorly regulated and escape supervision for multiple reasons.

### Traditional markets in developing countries

Traditional food markets flourish worldwide, serving as affordable food and nutrition sources and vital hubs for the informal economy. A review of the current literature indicates that the majority of these wet markets are located in developing regions [1,10, 16,17]. Due to their unique characteristics, these markets often present zoonotic and other health risks, as documented in various countries. These include numerous African nations [18,19], Asian countries such as Bangladesh [20,21], China [4,22–26], India [11], Thailand [27–29], Hong Kong [30–32], Indonesia [33], Myanmar [33], Malaysia [34,35], South Korea [36,37], Taiwan [38], Vietnam [39,40], Philippines [41] and many others [1,10,16,42]. While the specific features of these traditional food markets vary according to local preferences and existing conditions, their common characteristics, as outlined in the previous section, make them potential sources for the spread of zoonotic diseases and environmental health hazards.

### Putative risk factors

Due to their complex nature, traditional food markets present a variety of risks that carry significant public health implications. These risks and their associated factors can vary based on cultural differences and the specific operations of each market [2,4]. However, certain risk factors seem to be universally present, regardless of the setting.

#### Intermingling of different products

Due to the close proximity of various types of produce sold in traditional food markets, it is common for products to become intermingled. This characteristic facilitates the transfer of pathogens between products that would otherwise be separated by species barriers, such as meat and fish, origin, such as produce brought from different locations for sale with no other epidemiological linkages, and time, such as infected products brought early during trading that contaminate products brought and sold later. Tracing a specific risk becomes particularly challenging when products intermingle, for example, when vegetables are contaminated by a pathogen typically found only in meat.

#### Live animals from multiple species in crammed surroundings

Traditional food markets are known to deal in multiple species of live animals, which are brought from various places and are packed together for sale in areas with a high human population density. These conditions facilitate the transmission of pathogens between host species, potentially leading to the emergence of new zoonotic diseases.

#### Stress in animals

Animals brought to markets often experience significant stress due to a variety of factors, including inadequate facilities and space, as well as existing trading practices. This stress results in elevated cortisol levels. It is well documented that high cortisol levels can suppress the immune system and increase pathogen shedding. When these conditions are combined with the close quarters of densely packed cages, it creates an ideal environment for disease transmission.

#### In situ slaughter

The entrenched consumer preference for ‘fresh’ meat and fish often leads traders to slaughter live animals within their small premises and prepare the carcass on site. This practice not only results in unhygienic conditions, but also significantly increases the risk of disease transmission to a large population, including consumers, via contaminated products and aerosols, among other means.

#### No tracing-back of products

Tracing back products is virtually impossible due to the informal yet intricate networks involved in agricultural produce marketing, which typically lack any form of documentation. Often, the origins of products are obscured by multiple layers of traders who, fearing legal repercussions, are generally uncooperative. This is especially the case in markets that deal with wild animals sourced illegally and sold as traditional delicacies.

#### Eatables sold in the same premises

Many traditional food markets also feature shops that cater to the culinary needs of traders and buyers. However, the unhygienic conditions of these establishments, combined with the serving of food in close proximity to live animals, slaughtering activities, and raw products, present a significant risk for the transmission of foodborne diseases.

### Zoonotic disease risks

**Figure 1:**
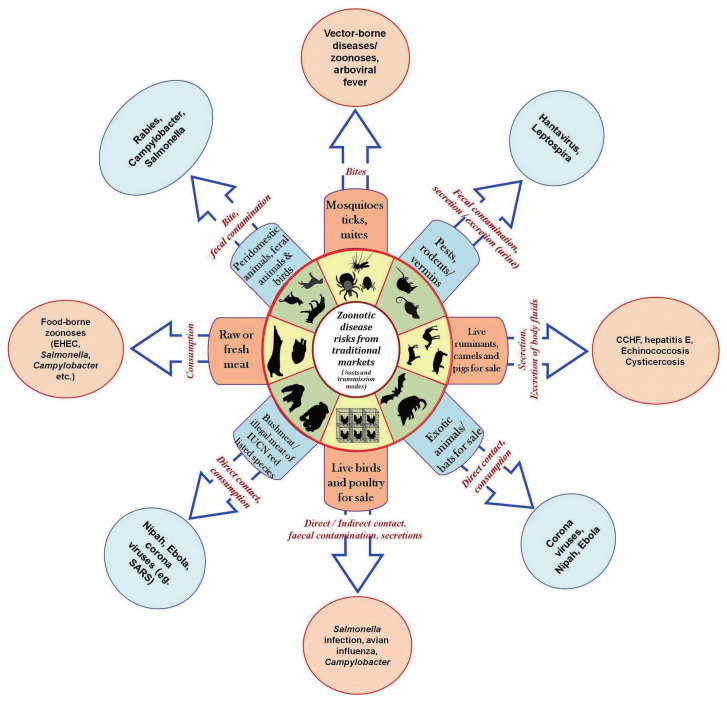
Risks of zoonotic diseases and potential routes of transmission from traditional markets. Blue arrows indicate modes of transmission. CCHF, Crimean-Congo hemorrhagic fever; EHEC, enterohemorrhagic *Escherichia coli*; SARS, severe acute respiratory syndrome; ICUN, International Union for Conservation of Nature.

Zoonotic and infectious diseases often emerge and reemerge due to a complex interplay of anthropogenic and non-anthropogenic factors, which can be difficult to predict [43,44]. Traditional food markets are often seen as potential “hot spots” for the emergence and spread of diseases due to the close proximity of a variety of agricultural products, including live animals, and the informal, lightly regulated nature of these markets [4]. Although it can be challenging to document, numerous zoonotic diseases have the potential to spread from these traditional food markets. The majority of these diseases are viral, but bacterial diseases can also spread. A variety of zoonotic diseases can be transmitted to humans from different animal species, as well as their secretions and excretions, through both direct and indirect routes (Figure 1). For more detailed information, readers are referred to Naguib et al. [4].

### Regulatory challenges for traditional markets

Traditional food markets are usually regulated by local governments. However, the various components of a value chain or food business often fall under different laws and authorities in many countries, which can complicate enforcement. Studies have detailed the complexities and intricacies of the legal tools available in different countries to address safety concerns in traditional wet food markets [10,17–19,41,45,46]. Therefore, regulating these markets is especially challenging, as the operational forces behind these markets often involve local consumer preferences, cultural practices, economic aspirations of neglected sectors of the population, potential health and environmental hazards, micro-scale societal well-being, and many others [4,8–10,16,40,47]. Despite these challenges, there have been sporadic attempts to gain a better understanding of these markets. For instance, Lin et al. [2] proposed a classification for wet markets that could be beneficial in shaping future regulatory frameworks. Past experiences with the harmonization of food regulations across numerous countries suggest that international organizations like the Codex Alimentarius Commission could play a crucial role [48]. However, regulating traditional markets is likely to present even greater challenges [49].

### Strategies for risk mitigation

In order to contain the public health risks from traditional food markets, a multipronged strategy is required. Given the complex and multifactorial nature of these risks, they must be addressed at various levels [1,2,4,8–10]. Major areas requiring attention are outlined below.

#### Regulatory framework

**Figure 2:**
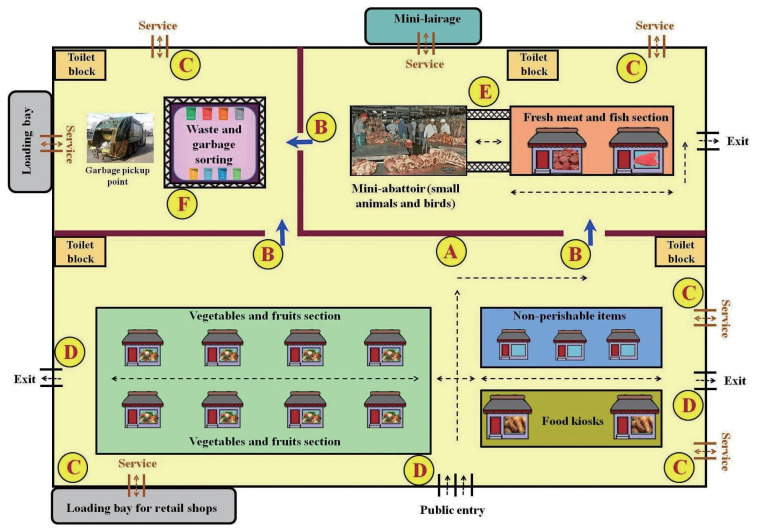
Proposed layout for a traditional market with hygienic features integrated into the design. The market should have the following features: (A) a clear separation between clean areas (such as food kiosks and non-perishable items) and unclean areas (like fresh meat and fish sections, and waste handling areas); (B) a one-way movement system that directs traffic from cleaner sections towards unclean sections; (C) designated entry and exit points exclusively for service personnel assigned to each section; (D) separate entrances and exits for the public to facilitate easy monitoring; (E) a movement path that is strictly limited to personnel responsible for transporting meat and fish from the mini-abattoir to the fresh meat and fish section; and (F) an additional barrier around the waste and garbage sorting facility to further prevent contamination.

There is a clear need for a unified regulatory framework to ensure the standards of hygiene, public health safety, trade, and environment-related regulations (e.g., source declaration, packaging requirements, waste disposal, etc.) are met in traditional food markets (Figure 2). This framework should encompass various aspects of construction and design standards, such as location, buyer direction control, product separation, stall placement, and entry control. Ideally, a single authority should be established to serve as a contact point for all other agencies, monitoring and enforcing relevant legislation. This mechanism would also facilitate information gathering and compliance, and aid in traceability during investigations of potential public health risks, including zoonotic and foodborne diseases.

#### Community-based risk management

Traditional food markets primarily serve as community assets, thus necessitating community involvement in risk mitigation strategies. Furthermore, it is crucial to educate primary producers, farmers, retailers, and traders, as well as to raise consumer awareness about market hygiene and the advantages of hygienic products.

#### Involvement of local governments and agencies

Local regulatory bodies continue to play a crucial role in maintaining safety within traditional food markets and their surrounding areas. However, it is imperative that these agencies are provided with up-to-date knowledge, tools, and practices. Additionally, their activities should be consolidated under a single, central agency that is specifically tasked with overseeing traditional food markets.

#### Economic, social, and cultural sensitivity

Traditional food markets thrive based on consumer preferences, which include the demand for fresh foods, affordability, proximity to residences, social bonding, and linguistic homogeneity, among others. Consequently, any mitigation strategies must take these factors into account to ensure they are both adoptable and practicable.

#### Scientific interventions

Preventing the spillover of zoonotic pathogens from traditional food markets necessitates scientific interventions. A framework for regular surveillance and monitoring of animal and human pathogens with potential for zoonotic transmission should be developed, preferably including pathogens not yet known to cause human diseases. Given the complex nature of traditional food markets, a One Health approach is ideally suited. For the quantitative estimation of the current situation and impact assessments of future programs, a Market Hygiene Index (MHI) should be developed. This index would combine various data points, such as market size, products dealt, infrastructure, waste disposal, people’s behavior, and weather conditions. The MHI could then be used to provide incentives to markets that exhibit desirable features.

#### Political commitment

No strategy or program can succeed without explicit political will. Therefore, it is essential to educate decision-makers about the potential public health risks associated with traditional food markets, as well as potential solutions to these risks.

Traditional wet food markets play a crucial role in societies worldwide, offering both benefits and risks. Despite numerous studies highlighting the potential zoonotic risks associated with these markets [1,16,42,50], systematic meta-analyses of traditional wet food markets are scarce, making evidence-based decision-making challenging. Consequently, our arguments are based on the available literature, which often lacks a comprehensive view. We acknowledge this as a limitation of our manuscript. Additionally, traditional wet markets are significantly shaped by local customs, preferences, and cultural settings, which complicates the task of generalization. Nevertheless, recent efforts [2] to define these markets represent a positive step forward.

## CONCLUSION

Traditional food markets are integral to societies worldwide, offering affordable food and nutrition to vast populations. However, due to their unique characteristics and the absence of standardized guidelines and approved designs, these markets are susceptible to public health risks, particularly the spillover of zoonotic pathogens. Consequently, the new design and MHI that we propose could serve as a significant barrier to these risks. When implemented alongside multilateral efforts adopting a One Health approach, these measures are likely to be particularly effective.

## Figures and Tables

**Figure f3-epih-45-e2023097:**